# N-Heterocyclic Carbene Monolayers on Metal-Oxide Films: Correlations between Adsorption Mode and Surface Functionality

**DOI:** 10.1021/acs.langmuir.4c01109

**Published:** 2024-05-03

**Authors:** Einav Amit, Rajarshi Mondal, Iris Berg, Zackaria Nairoukh, Elad Gross

**Affiliations:** † Institute of Chemistry, The Hebrew University, Jerusalem 91904, Israel; ‡ The Center for Nanoscience and Nanotechnology, The Hebrew University, Jerusalem 91904, Israel

## Abstract

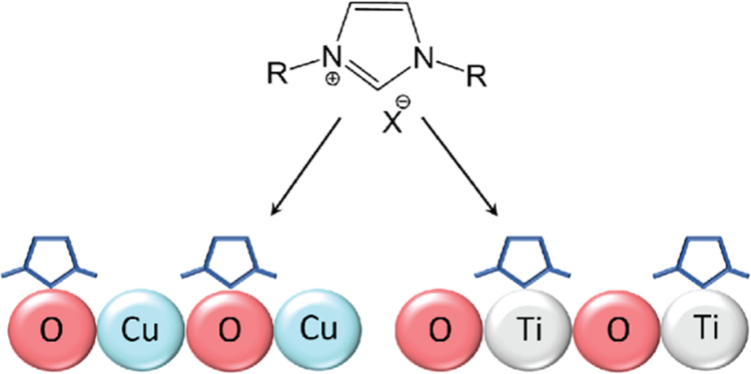

N-Heterocyclic carbene (NHC) ligands have been self-assembled on various metal and semimetal surfaces, creating a covalent bond with surface metal atoms that led to high thermal and chemical stability of the self-assembled monolayer. This study explores the self-assembly of NHCs on metal-oxide films (CuO*_x_*, FeO*_x_*, and TiO*_x_*) and reveals that the properties of these metal-oxide substrates play a pivotal role in dictating the adsorption behavior of NHCs, influencing the decomposition route of the monolayer and its impact on work function values. While the attachment of NHCs onto CuO*_x_* is via coordination with surface oxygen atoms, NHCs interact with TiO*_x_* through coordination with surface metal atoms and with FeO*_x_* via coordination with both metal and oxygen surface atoms. These distinct binding modes arise due to variances in the electronic properties of the metal atoms within the investigated metal-oxide films. Contact angle and ultraviolet photoelectron spectroscopy measurements have shown a significantly higher impact of F-NHC adsorption on CuO*_x_* than on TiO*_x_* and FeO*_x_* , correlated to a preferred, averaged upright orientation of F-NHC on CuO*_x_*.

## Introduction

N-Heterocyclic carbenes (NHCs) emerged as a robust and versatile alternative to thiols for the formation of self-assembled monolayers (SAM) on various surfaces.^1−8^ The carbene carbon is characterized with a strong σ-donor affinity, which is further stabilized by π back-donation from the metal,^1,2,4,9,10^ making it a strongly coordinated ligand to metal films and metallic nanoparticles.^1,11−16^ It was demonstrated that NHCs have higher binding energy to metals than thiols, leading to improved thermal and chemical stability of carbene-based monolayers.^11^ In addition, chemical functionalization of NHCs enabled the formation of various chemically addressable monolayers^17−20^ and their utilization in applications spanning from microelectronics to biosensors and corrosion mitigation.^19−31^

NHCs have been assembled on various metal surfaces, including Au, Ag, Pd, Pt, Ru, Mg, and Cu.^14,32−38^ In addition to the self-assembly of NHC monolayers on metal surfaces, it was shown that NHCs can self-assemble on hydrogen- and boron-terminated silicon.^39,40^ The Si–carbene bond was found to exhibit a predominant σ character, as indicated by the observed rotational freedom around the bond axis and the significantly low thermodynamic barrier calculated for such rotation.^33,39,40^

The self-assembly of NHCs on metal oxides is highly desired due to the significant role of metal oxides in material science and engineering,^41−46^ and the potential impact in monolayer functionalization for tailoring surface properties. A diverse range of organic precursors was previously employed for SAM formation on various metal oxides, including silica,^47,48^ alumina,^49,50^ and indium–tin oxide.^51,52^ SAM precursors can be broadly classified into two groups based on their binding mechanism: the first group involves binding via surface hydroxyls, while the second group binds to metal cations. The hydroxyl-binding group comprises silanes,^53−55^ phosphonates,^52,56−58^ and carboxylates,^59−61^ whereas the cation-binding precursors include ligands such as thiols and imidazolines.^51,62,63^

It was demonstrated that NHC monolayers can be self-assembled on oxyphilic substrates that are readily prone to oxidation, such as copper and magnesium films.^35,38,64,65^ Analysis of the self-assembly mechanism showed that during the self-assembly process, the NHCs reduced and etched the metal-oxide layer via NHC interaction with surface oxygen, enabling the formation of an NHC-metal bond.^35,66^ In both cases, NHC self-assembly was performed using a high concentration of solvated NHCs and a relatively thin oxide layer that facilitated metal-oxide reduction.

Recent studies have shown that under ultrahigh-vacuum (UHV) conditions, NHCs can directly bind to copper-oxide surfaces without reduction of the metal-oxide layer.^67,68^ It was identified that the NHC binding mode on copper oxide was via surface oxygen rather than the metal atoms.^67,68^ DFT simulations quantified an adsorption energy gain of 1.16 eV for NHC adsorption on oxygen vs Cu in the CuO*_x_* layer, demonstrating the thermodynamic preference for carbene adsorption on oxygen surface atoms. However, it should be noted that in this example, the NHC deposition was performed under UHV conditions with exposure to gas-phase carbene molecules. These conditions minimized the surface concentration of carbenes and their reductive influence on surface properties.

This study aims to demonstrate the feasibility of NHC self-assembly on metal oxides under liquid phase conditions and identify the role of metal oxides in dictating the adsorption behavior of NHCs and the impact of NHC monolayers on surface properties. Electrochemical deposition was employed for deprotonation of the imidazole precursor for NHC formation.^37^ This deposition method induces a local increase in the concentration of NHCs in high proximity to the metal-oxide surface, thus minimizing the reductive effect of NHCs on the metal-oxide film. NHCs were electrochemically deposited on CuO_*x*_, FeO_*x*_, and TiO_*x*_, and the influence of NHC monolayers on surface hydrophobicity and work function values were quantified. It was identified that NHCs were anchored on the CuO_*x*_ film via surface oxygen atoms, whereas their binding to FeO_*x*_ and TiO_*x*_ was mostly through metal atoms. Contact angle and work function measurements have shown a higher impact of NHC adsorption on CuO_*x*_, correlated with a more upright orientation of NHCs on this surface.

## Results and Discussion

**Figure 1:**
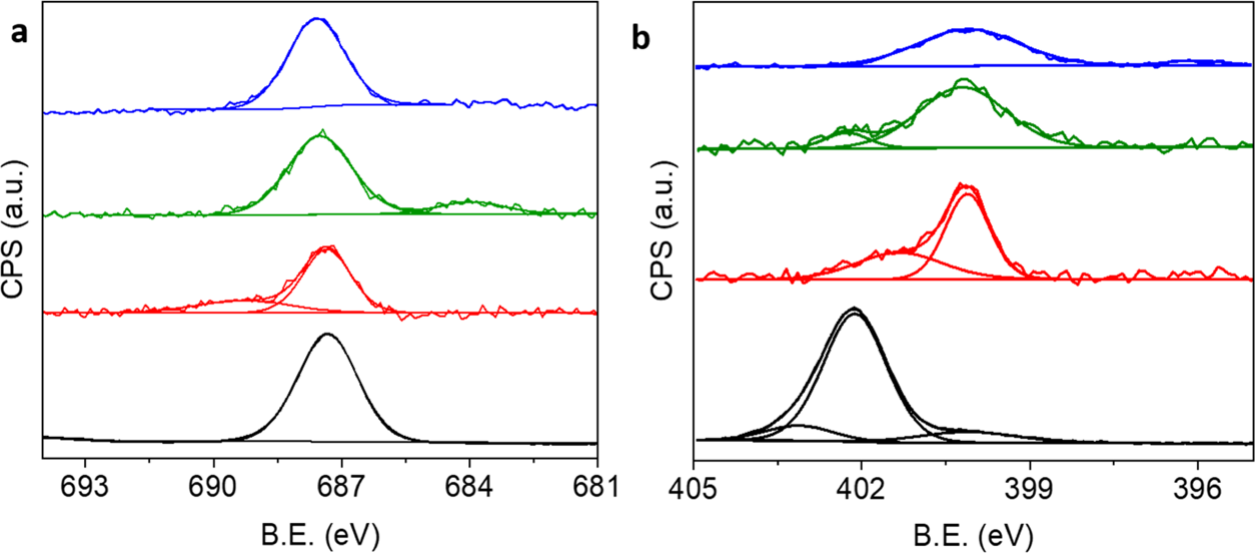
F 1s (a) and N 1s (b) XPS signals of the imidazolium salt precursor (black-colored) and following F-NHC electrodeposition on CuO_*x*_, FeO_*x*_, and TiO_*x*_ films (red-, green-, and blue-colored spectra, respectively).

**Scheme 1:**
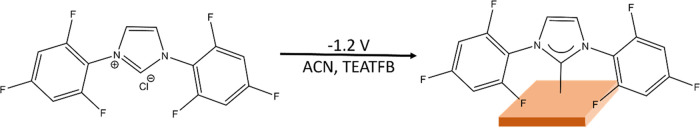
Electrochemical Deposition of F-NHC on Metal-Oxide Films ACN, acetonitrile; TEATFB, tetraethylammonium tetrafluoroborate.

Fluorine-functionalized imidazolium salt was synthesized and self-assembled on CuO_*x*_, FeO_*x*_, and TiO_*x*_ films by electrochemical deposition (Scheme 1, see the Experimental Section for additional information).^37,69,70^ The fluorine atoms in fluorine-functionalized NHCs (F-NHC) are used as indicators for NHC adsorption by probing their F 1s X-ray photoelectron spectroscopy (XPS) signal (Figure 1). The presence of nitrogen residues in some of the metal-oxide films made it necessary to use this additional marker for probing the self-assembly of the NHCs. NHC fluorination is expected to induce a relatively minor impact on the electronic properties of the metal oxide and does not noticeably change the surface density of NHCs.^71^

The F 1s XPS signal of the imidazolium salt precursor showed a single peak at 687.3 eV (Figure 1a, black-colored spectrum). F-NHC monolayers on the various metal oxides showed a similar F 1s XPS signal at 687.4 eV. For comparison, the F-NHC monolayer was also electrodeposited on Au film and showed a similar peak pattern with a Gaussian fit at 687.4 eV (Figure S1). The F 1s XPS signal on CuO_*x*_ showed an additional minor peak at 689.3 eV, correlated to an oxidized fluorine species.^72,73^ A minor spectral feature was detected at 684.5 eV in the F 1s XPS signal of F-NHC on FeO_*x*_ (Figure 1), attributed to fluorine that strongly interacts with metal cations^74,75^ with a characteristic of semi-ionic fluorine species.^76^

It should be noted that the imidazolium salt precursor was easily removed from the surface following rinsing, without any trace of the F 1s XPS signal (Figure S2). Thus, the detection of F 1s XPS signal, following electrodeposition and rinsing, can indicate the chemisorption of the F-NHC monolayer on the surface. It was previously shown that the F 1s XPS signal of thiol-based monolayers with a high density of CF_3_ groups is characterized with a binding energy of ∼688 eV,^77^ while the F 1s XPS signal of F-NHC monolayers on Au and metal oxides was centered at 687.4 eV. The variations in peak position may be attributed to the coordination of the fluorine to the phenyl ring in F-NHC and its high proximity to the surface.

The atomic percentages of fluorine on copper oxide, iron oxide, titanium oxide, and gold films were 0.6 ± 0.1, 1.1 ± 0.2, 2.7 ± 0.4, and 3.0 ± 0.6%, respectively. These results indicate that a denser F-NHC monolayer was formed on the Au film, which can be attributed to an optimized NHC adsorption on the fully metallic Au surface.^78^ Analysis of the oxygen-to-metal atomic ratio revealed that NHC deposition did not change the oxidation state of the metal-oxide films (Table S1).

The N 1s XPS spectrum of the imidazolium salt precursor (Figure 1b, black-colored spectrum) was centered at 401.8 eV, which is similar to previously described imidazolium salt precursors.^20^ An additional minor peak was identified at 399.9 eV and correlated to the presence of impurities, originating from the synthesis of the imidazolium salt precursor. Following deprotonation and surface anchoring of F-NHC on metal-oxide films, the main peak shifted to lower binding energies and was centered at 400.1, 400.2, and 400.1 eV for F-NHC on CuO*_x_*, FeO*_x_*, and TiO*_x_*, respectively. The shift in the peak position was correlated to the neutralization of the positive charge and was identified in various NHC-based monolayers.^79^ Additional minor peaks were detected at 401.8–402.0 eV. These peaks can be attributed to the pyrrolic N=C bond, commonly observed after partial decomposition of the imidazole ring.^36,80^ Similar peaks were observed for protonated pyrroles or N–O bonds.^81,82^ The high-energy features in the N 1s XPS signals were not observed following annealing to 100 °C, which indicates that the origin of this spectral signature arises from physisorbed molecules, while the dominant peak at 400.1–2 eV was still detected, as expected for chemisorbed species. It should be noted that the wider N 1s XPS peak for F-NHC on TiO_*x*_ wasinduced due to a nitrogen signal that originated from the TiO_*x*_ sample and can be attributed to the presence of TiN. Survey XPS spectra following deposition of F-NHC on metal oxides were conducted and mostly revealed the signature of F-NHC on the surface with no indication of physisorbed halide or physisorbed electrolyte residues (Figure S3). It was demonstrated that electrolyte residues do not reside on the surface following electrochemical deposition of NHCs and postdeposition rinsing.^37^

The metal-oxide surface roughness was analyzed both before and after deposition of F-NHC monolayers by atomic force microscopy (AFM) measurements (Figure S4). The roughness of CuO_*x*_, FeO_*x*_, and TiO_*x*_ prior to NHC deposition was 13 ± 1, 0.9 ± 0.2, and 1.7 ± 0.1 nm, respectively. Following electrodeposition of F-NHC, the roughness of CuO_*x*_, FeO_*x*_, and TiO_*x*_ was 22 ± 5, 0.9 ± 0.3, and 2.2 ± 0.2 nm, respectively. These results show that corrugated surfaces were more prone to corrugation changes following F-NHC deposition. X-ray diffraction (XRD) spectra of metal-oxide films were measured to identify their crystallinity (Figure S5). No dominant diffraction peaks were identified for CuO_*x*_ and TiO_*x*_, and minor Fe_2_O_3_ diffraction peaks were detected for FeO_*x*_. These results indicate that the thin metal-oxide films do not form a well-defined crystalline phase.

**Figure 2:**
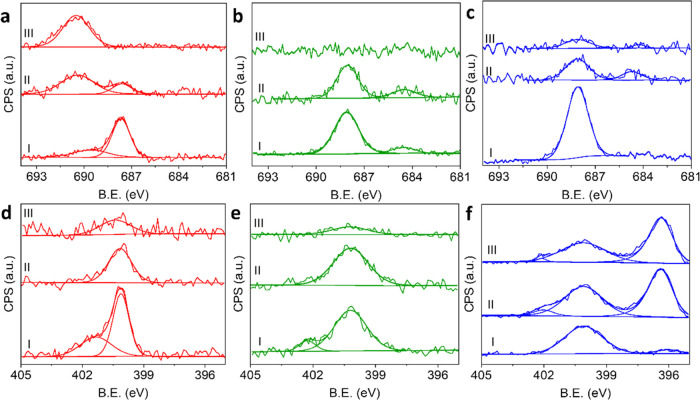
F 1s (a–c) and N 1s (d–f) XPS measurements of F-NHC on CuO_*x*_ (a, d), FeO_*x*_ (b, e), and TiO_*x*_ (c, f) at room temperature (I), after annealing to 100 °C (II) and 200 °C (III).

The thermal stability of the monolayers was evaluated by annealing the NHC-coated metal oxides to 100 and 200 °C (2 h, under air) and measuring the resulting F 1s and N 1s XPS signals (Figure 2a–c,d–f, respectively). Analysis of the N 1s XPS signals showed that annealing of the samples to 100 °C led to desorption of the physisorbed species, as identified by the disappearance of the high-energy feature in the N 1s XPS spectra of CuO_*x*_ and FeO_*x*_ (Figure 2d,e, respectively), while more minor changes in the peak pattern were detected for F-NHC on TiO_*x*_ (Figure 2f). Annealing to 200 °C led to a decrease in the N 1s XPS peak area for all three samples, thus indicating that desorption occurs at this temperature on all three metal-oxide surfaces. It should be noted that desorption can also be coupled with partial decomposition and fragmentation of the surface-anchored NHCs.

A dominant N 1s XPS signature was detected on TiO_*x*_ after annealing to 200 °C (Figure 2f). An additional N 1s XPS peak was detected at 396.3 eV following annealing to 100 °C and was attributed to nitrogen bulk contamination that diffused to the surface during annealing. This peak can be related to the formation of TiN.^83^ Control experiments, in which TiO_*x*_ was annealed without F-NHC monolayer deposition, were performed and led to an increase in the amplitude of the N 1s XPS peak at 396.3 (Figure S6). Moreover, the TiO_*x*_ sample was also characterized with a background N 1s XPS signal at ∼400 eV. Therefore, it cannot be excluded that the nitrogen XPS signal on TiO_*x*_ partially originated from nitrogen bulk-to-surface diffusion.

Based on the temperature-dependent F 1s XPS spectra, it is possible to identify potential decomposition routes induced by C–H, C–N, or C–F bond dissociation following exposure to elevated temperature. C–H bond dissociation should not noticeably affect the core level bonding energies of F, while a C–F bond dissociation should lead to a peak ∼5 eV lower than the bands corresponding to F bonded to C. NHC decomposition via C–N bond dissociation can induce better interaction between the F-phenyl group and the surface atoms and thus lead to a shift toward higher binding energies of F, with relatively small changes in the overall F 1s peak area.

At room temperature, the F 1s XPS spectrum of F-NHC on CuO_*x*_ included a dominant peak at 687.3 eV and a minor peak at 689.3 eV (Figure 2a). Sample annealing led to a continuous decrease in the low-energy peak and an increase in the high-energy peak. After annealing to 200 °C, a single peak was detected at 690.4 eV. The N 1s XPS peak of F-NHC on CuO_*x*_ was located at 400.0 eV and continuously decreased following annealing (Figure 2d). Thus, surface annealing of F-NHC on CuO_*x*_ did not noticeably modify the total F 1s peak area but led to a shift in the peak position coupled with a decrease in the N 1s XPS signal. These results indicate that exposure of F-NHC on CuO_*x*_ to elevated temperatures led to molecular fragmentation, induced by C–N bond cleavage and carbene ring desorption, while the fluorinated side groups were firmly anchored to the surface and did not desorb even after annealing to 200 °C.

A different deformation pattern was obtained for F-NHCs that were self-assembled on FeO_*x*_, in which a continuous decrease in both the F 1s and N 1s XPS signals was detected following annealing (Figure 2b,e, respectively). An additional minor feature was detected in the F 1s spectra at 684.5 eV (Figure 2b), attributed to fluorine that strongly interacts with metal cations.^74,75^ The temperature-dependent F 1s XPS peak pattern for F-NHC on TiO_*x*_ was similar to that of FeO_*x*_ and was correlated, as well, to the desorption of F-NHC rather than decomposition. A sharper decrease in the F 1s XPS peak amplitude was detected on TiO_*x*_, compared to FeO_*x*_, which can be assigned to weaker F-NHC interactions on this surface. It can be, therefore, concluded that variations in NHC-substrate interactions influenced the desorption and decomposition routes of surface-anchored NHCs.

Oxygen-to-metal atomic ratios were slightly modified following annealing of F-NHC-coated FeO*_x_* and TiO*_x_* substrates (Table S1). A decrease was obtained in the oxygen-to-metal ratio of F-NHC on CuO*_x_* and was attributed to the desorption of NHC molecules that were chemisorbed to surface oxygen atoms, as previously described.^66^

Quantitative analysis of the atomic percentage of fluorine atoms on the metal-oxide films showed that the smallest change in surface concentration of fluorine atoms was identified for CuO_*x*_, in which a 40% decrease in the atomic concentration of F was measured after annealing to 100 °C (Table S2). A decrease of 70 and 60% in the atomic concentration of fluorine was detected for F-NHC on FeO*_x_* and TiO*_x_*, respectively, after annealing to 100 °C. The variation in surface density of F on the different metal oxides was correlated with the changes in the dissociation pattern. C–N bond cleavage and surface adsorption of F-phenyl were identified on CuO*_x_*, while F-NHC desorption, potentially coupled with decomposition, was facilitated on FeO*_x_* and TiO*_x_*. Continuous exposure of the NHC monolayer to the X-ray beam did not lead to noticeable changes in the F 1s XPS peak pattern, indicating that no beam-induced damage leads to dissociation or decomposition of F-NHCs (Figure S7).

**Figure 3:**
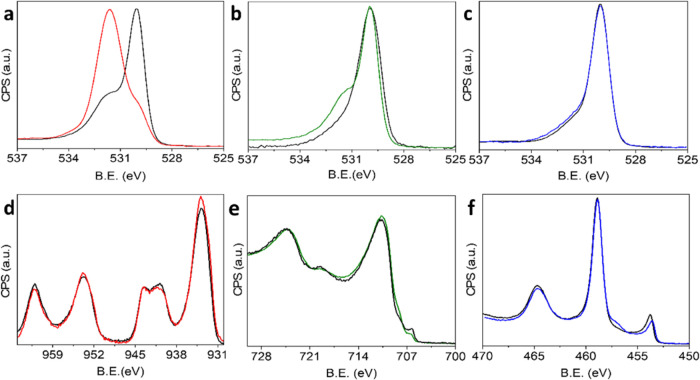
O 1s XPS signals of CuO_*x*_ (a) FeO_*x*_ (b), and TiO_*x*_ (c) and XPS signals of Cu 2p (d), Fe 2p (e), and Ti 2p (f) were measured before (black-colored) and after (red-, green- and blue-colored, respectively) self-assembly of F-NHC.

The metal and oxygen XPS signals were analyzed to identify the cause for the variations in surface density and decomposition route of NHCs on the different metal oxides (Figure 3). Prior to NHC deposition, the O 1s XPS signal of CuO_*x*_ was constructed of a main peak at 530.1 eV and a minor peak at 531.6 eV (Figure 3a, black-colored spectrum). The low-energy peak was correlated to oxygen in the metal-oxide layer,^84,85^ while the smaller, high-energy peak was correlated to the oxygen–carbon bond, potentially originating from carbonates and hydroxide species on the surface, and also to the presence of surface-bound hydroxyl species (metal(OH)*_x_*).^86,87^

Following NHC deposition, the XPS pattern was utterly changed, and the high-energy peak became dominant, while the low-energy-peak amplitude decreased (Figure 3a, red-colored spectrum). The Cu 2p XPS signal did not show noticeable changes following NHC anchoring (Figure 3d). The XPS spectra therefore show that NHCs were anchored on CuO_*x*_ via oxygen atoms, as previously identified for vapor-phase deposition of NHCs on CuO_*x*_ under UHV conditions.^67,68^ Temperature-dependent XPS measurements identified that annealing of F-NHC-coated CuO_*x*_ led to the imidazole ring desorption (Figure 2) along with variations in the O 1s signal pattern (Figure S8). The temperature-dependent changes in the XPS signal further validated the connection between the O 1s spectral variations and F-NHC adsorption and that thermal annealing led to NHC fragmentation and carbene ring desorption.

The O1s XPS spectrum of FeO_*x*_ showed milder changes following F-NHC adsorption, with a shoulder detected at 532.0 eV (Figure 3b). A shoulder was also probed at 708.2 eV in the Fe 2p XPS spectra following F-NHC deposition (Figure 3e). A similar signal at 708 eV was correlated to Fe–C species and is therefore indicative of strong interaction with surface metal atoms.^88,89^ Temperature-dependent XPS measurements showed that following F-NHC desorption, the O 1s and Fe 2p XPS spectra were modified, and features that were related to NHC adsorption, at 532 eV for the O 1s and 708.2 eV for the Fe 2p, were not observed following annealing (Figure S8). The changes in both metal and oxygen XPS signatures correlate to NHC desorption from FeO_*x*_, which is similarly evident in the N 1s and F 1s XPS spectra (Figure 2).

NHC adsorption on TiO_*x*_ did not affect the O 1s XPS spectrum (Figure 3c). However, a shoulder at 456.9 eV was detected in the Ti 2p XPS signal, following NHC deposition (Figure 3f). The signal at 456.9 eV is close to the shoulder obtained after sulfur adsorption on TiO_*x*_, in which the sulfur replaced the oxygen atom in the oxide lattice.^90^ These spectral changes indicate that F-NHCs were mostly self-assembled on TiO_*x*_ via an interaction with the metal atoms.

Analysis of the C 1s XPS signals on the three metal oxides before and after F-NHC deposition shows an overall increase in the carbon signature, which can rise from the presence of F-NHC and solvent residues (Figure S9). The C 1s spectra of TiO*_x_* showed a larger increase in the C 1s XPS peak amplitude with a dominant shoulder at 286.6 eV, corresponding to the C–OH group, which may have originated from the formation of hydroxyls on the surface of TiO*_x_* during electrodeposition.

Grazing angle XPS measurements were performed for F-NHC on CuO_*x*_ (Figure S10). The main difference in the grazing angle measurements was obtained in the N 1s XPS spectrum that did not show the high-energy tail that was detected at a normal angle XPS measurement. This difference can be correlated to higher sensitivity toward chemisorbed NHCs that reside in high proximity to the surface.

Thus, the XPS data show that F-NHCs were anchored via surface oxygen atoms on CuO_*x*_ and metal surface atoms on TiO_*x*_, while F-NHC adsorption on FeO_*x*_ was conducted via both metal and oxygen surface atoms. It should be noted that the self-assembly of F-NHCs did not noticeably change the oxygen-to-metal atomic ratio (Table S1). Thus, the changes in the XPS signatures following NHC self-assembly were correlated to variations in surface binding modes and were not induced by oxidation or reduction of the metal-oxide films.

The differences in the binding modes of NHCs on metal oxides can be attributed to variances in the electronic configuration of the metal cations in the lattice. The surface of titanium oxide is expected to be the most reactive toward NHC adsorption due to the charge of titanium cations and their empty d orbitals, which are expected to have a strong affinity toward the carbene. The iron cations in iron oxide can have a charge of +2 and +3, depending on the crystal structure, and their d orbitals are partially filled ([Ar]3d^6^ for Fe(II) and [Ar]3d^5^ for Fe(III)). The copper cations have a maximal charge of +2, and their electronic configuration is [Ar]3d^9^, and are thus expected to have a lower affinity to NHCs.

Preferential binding through the titanium cation, which arises from its empty d orbitals, will facilitate the formation of a strong coordinative bond with ionic character. In contrast, the copper cation is relatively electron-rich and, therefore, exhibits a lower affinity to the carbene moieties compared with the electronegative oxygen or surface-bound hydroxyl groups. The binding affinity of iron-oxide cations to the carbene moieties depends on their cationic charge state. Fe(III) is expected to behave similarly to Ti(IV) cations, while Fe(II) is anticipated to show a lower affinity toward carbene adsorption in comparison to that of oxygen. Nevertheless, even for Fe(III), the binding is expected to be weaker than that of titanium oxide due to the partially filled d orbitals of Fe(III) that give rise to a more covalent nature.

The observed variations in the F-NHC binding modes on metal oxides can rationalize the differences in surface density. The highest surface density of F-NHCs was detected on TiO_*x*_ on which the NHCs strongly interact via metal surface atoms. The lowest surface density of F-NHC was measured on CuO_*x*_, in which the NHC is adsorbed via oxygen surface atoms.

**Figure 4:**
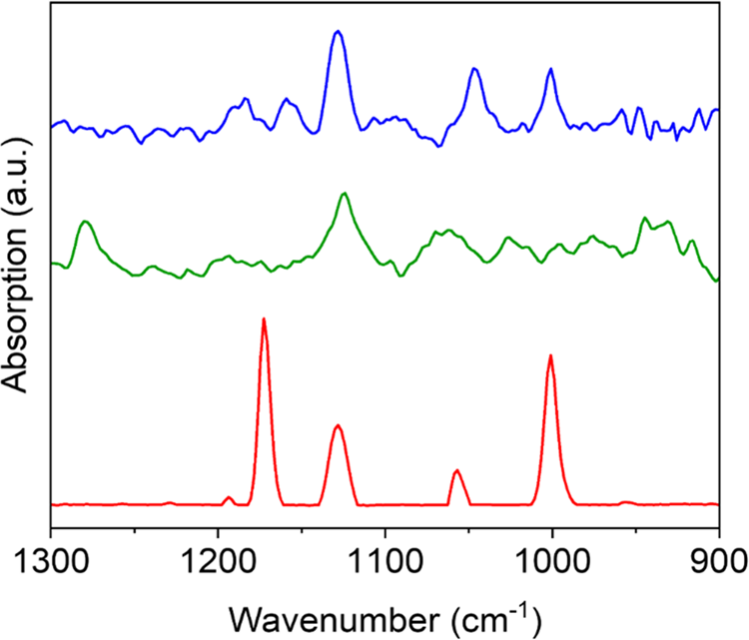
PM-IRRAS spectra of F-NHC monolayers on CuO_*x*_ (red), FeO_*x*_ (green), and TiO_*x*_ (blue).

An additional indication for variations in the NHC binding mode on different metal oxides was probed by a comparative analysis of the vibrational signature of F-NHCs on metal oxides by polarization modulation infrared reflection absorption spectroscopy (PM-IRRAS) measurements. Vibrational signatures were probed between 1000 and 1300 cm^–1^ and correlated to C–F bonds (Figure 4).^91^ Specifically, the vibrational peaks at 1000 and 1060 cm^–1^ were associated with C–F stretches, as previously identified for fluorinated aromatic compounds.^83^

The PM-IRRAS signals of F-NHC on metal oxides were shifted compared to the imidazolium salt precursor (Figure S11), correlated to the deprotonation and surface anchoring of NHCs. Thus, a direct comparison of the vibrational spectra of the NHC precursor and the surface-anchored NHC is not trivial and a complete peak assignment of the surface-anchored F-NHC will require computational analysis. A dominant vibrational signature was probed at 1172 cm^–1^ for F-NHC on CuO*_x_* and was associated with C–O vibrations, which can result from NHC binding to oxygen surface atoms. The intensity of this vibration decreased after annealing to 100 °C, and completely disappeared after annealing to 200 °C (Figure S12). The temperature-dependent changes indicate that this vibration is correlated to F-NHC adsorption on CuO_*x*_. Other vibrational signatures, located at 1000, 1060, and 1140 cm^–1^, which were correlated to C–F stretches, were detected after annealing to 100 °C and most of these peaks, except for the one at 1140 cm^–1^, were also probed after annealing to 200 °C. This result further supports the decomposition and fragmentation route, which was suggested based on XPS data.

A similar vibrational signature at 1176 cm^–1^ was also observed in the spectrum of the NHC monolayer prepared by deprotonation of dimethyl benzimidazolium iodide on CuO_*x*_. The appearance of the same vibrational signature in these NHCs can potentially indicate that this vibration is not related to the fluorinated phenyl moiety (Figure S13). It should be noted that the C–O vibration was not detected once the F-NHC monolayer was self-assembled on the Au film (Figure S14). These results reveal that the vibration at 1176 cm^–1^ was not correlated to the C–F bond and occurs only when the NHC is adsorbed on a metal-oxide film, thus further pointing out that it is a surface-induced C–O vibration. The C–O vibration was not probed in the spectra of F-NHC on TiO_*x*_ and FeO_*x*_, in which F-NHC anchoring was mostly induced via surface metal atoms (Figure 4). The lack of vibrational signal at 1000 cm^–1^ and the lower overall C–F vibration signals for F-NHC on FeO*_x_* can be attributed to the C–F dissociation route, which was detected in the F 1s XPS spectra following surface anchoring of F-NHC (Figure 2).

Contact angle measurements revealed that NHC adsorption led to an overall increase in the water contact angle values (Tables 1 and S3). The changes in the contact angle values induced by NHC adsorption on metal oxides were smaller than those measured on Au and correlated to the lower surface density of NHCs on metal oxides.^36,92,93^ The F-NHC monolayer on CuO_*x*_ induced the most significant increase in hydrophobicity, followed by FeO_*x*_ and TiO_*x*_. This observation is also consistent with the PM-IRRAS data that showed the highest signal-to-noise level for F-NHC on CuO*_x_*, which is indicative of an upright-oriented fluorinated monolayer and is predicted to form a more hydrophobic layer than a flat-lying one.^94^ The presented results therefore identify the averaged adsorption geometry of F-NHC based on PM-IRRAS measurements. A detailed analysis of the local ordering of the surface-anchored NHCs will require scanning tunneling microscopy (STM) measurements on well-defined surfaces, which is beyond the scope of this work.

**Table 1:** Contact Angle and Work Function Following F-NHC Self-Assembly

	Δcontact angle	Δwork function (eV)
CuO_*x*_	26 ± 4°	–0.27 ± 0.03
FeO_*x*_	15 ± 4°	0.09 ± 0.03
TiO_*x*_	12 ± 3°	0.06 ± 0.03

The changes in the work function values of metal oxides following self-assembly of F-NHCs were measured by ultraviolet photoelectron spectroscopy (UPS) (Table 1 and Figure S15). F-NHC monolayer formation lowered the work function of CuO_*x*_ by 0.27 eV, a slightly lower change compared to the impact of NHC monolayers on gold surfaces.^20,25,29^ In contrast, F-NHC monolayers on TiO_*x*_ and FeO_*x*_ induced a marginal increase in the work function. This also points to differences in the dipole moment induced by NHC monolayer,^20,40,95^ which further supports the observation of two distinct binding modes of NHCs to metal oxides.

## Conclusions

Self-assembled monolayers of F-NHC were prepared on CuO_*x*_, FeO_*x*_, and TiO_*x*_ films. Distinct binding modes were identified on different metal oxides, with F-NHC anchoring via surface oxygen atoms on CuO_*x*_, and via surface metal atoms on TiO_*x*_, while both binding modes were probed on FeO_*x*_. The variations in the binding modes influenced the surface density and dissociation patterns of NHCs. The lowest surface density was probed on CuO_*x*_, where F-NHC adsorption was conducted via surface oxygen atoms. C–N bond dissociation was induced by annealing and led to imidazole ring desorption, while F-phenyl was chemisorbed on the surface. The highest surface density was identified on TiO_*x*_, in which the F-NHC adsorption was coordinated via surface metal atoms. The differences in the binding modes were correlated to changes in the electronic configurations of the metal cations in the metal-oxide films. Contact angle and UPS measurements have shown a significantly higher impact of F-NHC adsorption on CuO_*x*_ than on TiO_*x*_ and FeO_*x*_, correlated to a more upright orientation of F-NHC on CuO_*x*_, which is the result of weaker ligand-substrate interactions. The presented results therefore demonstrate the influence of metal-oxide properties on the adsorption mode, surface density, and dissociation pattern of F-NHC monolayers and their consequential impact on surface properties.

## Experimental Details

Ti, Fe, and Cu films (100 nm thick) were evaporated on a highly doped n-type Si wafer with a 15 nm thick chromium adhesion layer. The coated Si wafers (2 cm × 1 cm) were thoroughly rinsed, dipped into 30% H_2_O_2_ for 2 min, rinsed with DI water and isopropanol, and dried under nitrogen before the deposition of NHCs.

Fluorinated NHC (F-NHC) was synthesized according to a published procedure.^96^

The coated Si wafers were placed in deposition solutions containing the fluorinated NHC imidazolium salt (5 mM), acetonitrile, an electrolyte (tetraethylammonium tetrafluoroborate, 0.1 M), and 55 mM water.^37^ Electrochemical (EC) deposition was conducted using a potentiostat (BioLogic) in a conventional three-electrode cell, with the Si wafer as the working electrode, Ag/Ag^+^ (CH Instruments) as a nonaqueous quasi-reference electrode, and a platinum wire as a counter electrode. The sample was held at a voltage of −1.2 V vs Ag/Ag^+^, which was applied in pulses for 10 min.^97,98^ Following pulsed electrochemical deposition, the sample was copiously rinsed with water and acetonitrile and dried under nitrogen.

X-ray photoelectron spectroscopy (XPS) measurements were performed using a Kratos AXIS Supra spectrometer (Kratos Analytical) with Al Kα monochromatic X-ray source (1486.6 eV). The XPS spectra were acquired with a takeoff angle of 90° (normal to analyzer), pass energy of 20 eV, and step size of 0.1 eV at 2 × 10^–9^ Torr. The binding energies were calibrated according to the C 1s XPS peak position (B.E. = 285.0 eV). Data were collected and analyzed using the ESCApe processing program (Kratos Analytical) and Casa XPS. The peaks were fitted with Gaussians with FWHM < 1.8. Quantitative data, such as atomic percentages, was obtained directly from the XPS measurements, without further analysis.

Polarization modulation infrared reflection absorption spectroscopy (PM-IRRAS) measurements were performed at room temperature under positive nitrogen pressure in a reflection–absorption cell (Harrick, Inc.) with a PM-FTIR spectrometer (PMA-50 coupled to a Vertex 70, Bruker). Measurements were performed using 1024 scans at a resolution of 4 cm^–1^ with a mercury cadmium telluride (MCT) detector.

The UPS spectra were obtained using a Kratos Axis Supra spectrometer (Kratos Analytical), with a HeI discharge lamp (excitation energy of 21.22 eV) as the ultraviolet source. Measurements were conducted at a 0.025 eV resolution range of 20 to −5 eV. The accumulation time was 180 s. The vacuum was maintained at 2 × 10^–9^ Torr. Photoelectrons generated from the UV radiation were collected and analyzed in the same manner as in the XPS; due to the low kinetic energy of the UV-induced electrons in the UPS, the lenses were switched to high-precision and low-voltage outputs to provide the necessary accuracy in the low-voltage UPS regime. The work function values were calculated by fitting the secondary electron cutoff from the UPS spectra.^99^

Contact angle measurements were performed using a Rame-Hart 100 goniometer (Rame-Hart Instrument) equipped with an automated dispensing system (5 μL drops).

## Supporting Information Available

Additional XPS, UPS, and PM-IRRAS measurements of metal oxides with and without F-NHC monolayers (PDF)
