# Supplementary material for: N-Heterocyclic Carbene Monolayers on Metal-Oxide Films: Correlations between Adsorption Mode and Surface Functionality
Source: Langmuir. 2024 May 3;40(19):10374–83. doi: 10.1021/acs.langmuir.4c01109 (PMC11100006; doi:10.1021/acs.langmuir.4c01109)
Supplement: Supplementary file 1 — la4c01109_si_001.pdf [file la4c01109_si_001.pdf]

Supporting Information

N-Heterocyclic Carbene Monolayers on Metal Oxide Films: Correlations Between Adsorption Mode and Surface Functionality

Einav Amit[1,2], Rajarshi Mondal[1], Iris Berg[1,2], Zackaria Nairoukh[1] and Elad Gross[1,2]*

1 Institute of Chemistry, The Hebrew University, Jerusalem 91904, Israel

2 The Center for Nanoscience and Nanotechnology, The Hebrew University, Jerusalem 91904, Israel

* Corresponding author email address: elad.gross@mail.huji.ac.il

Table S1: Oxygen to metal atomic ratio as identified based on XPS analysis

	CuO_x	FeO_x	TiO_x
Metal-oxide	1.6±0.1	2.6±0.3	2.5±0.2
F-NHC coated metal-oxide	1.6±0.1	2.5±0.2	2.3±0.2
F-NHC coated metal-oxide (annealed to 100 °C)	1.8±0.1	2.8±0.2	2.1±0.2
F-NHC coated metal-oxide (annealed to 200 °C)	1.2±0.1	2.4±0.2	2.1±0.2

Table S2. Relative atomic percentage of F as quantified by XPS analysis

	**Cu**	**Fe**	**Ti**
RT	1	1	1
100°C	0.6±0.1	0.3±0.1	0.4±0.1
200°C	0.2±0.1	0.0	0.10±0.03

Table S3: Absolute contact angle and work function values

.

	**Work function (eV)**	**Contact angle (°)**
CuOx	4.43	16±4
F-NHC/CuOx	4.16	42±3
FeOx	4.20	67±3
F-NHC/FeOx	4.29	82±4
TiOx	3.99	41±2
F-NHC/TiOx	4.05	53±3

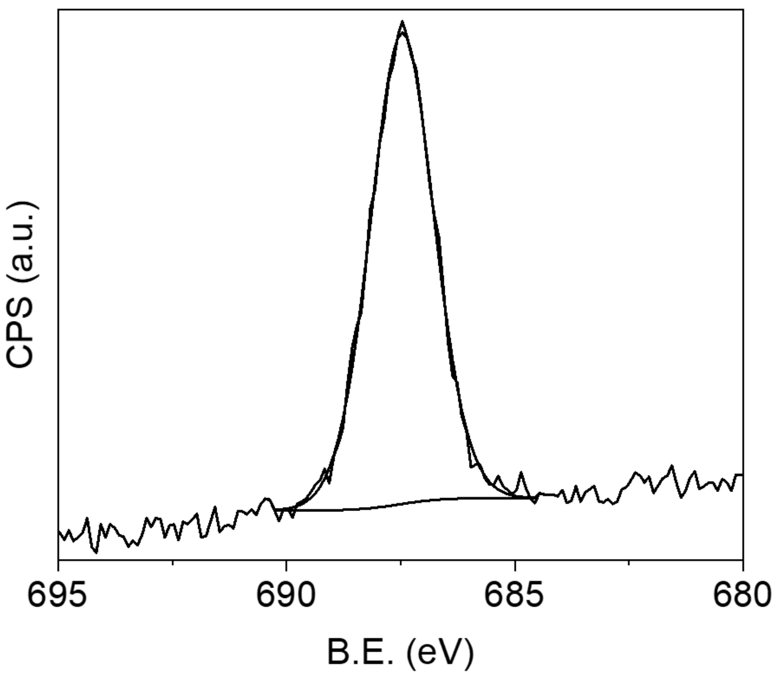

Figure S1: F1s XPS signal of F-NHC on Au film.

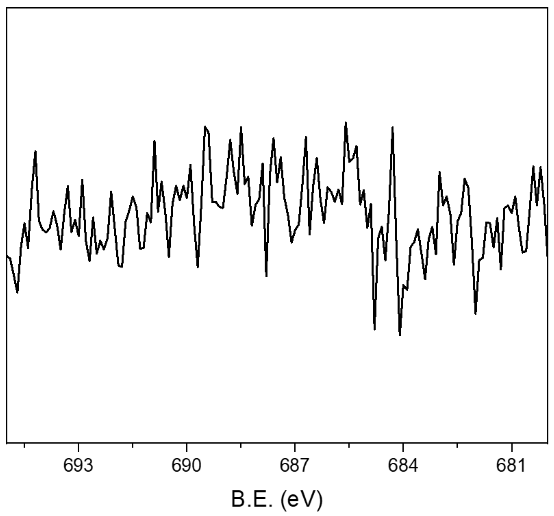

Figure S2: F1s XPS spectra measured after deposition of solvated imidazolium salt precursor on CuOx, followed by rinsing in water and acetonitrile.

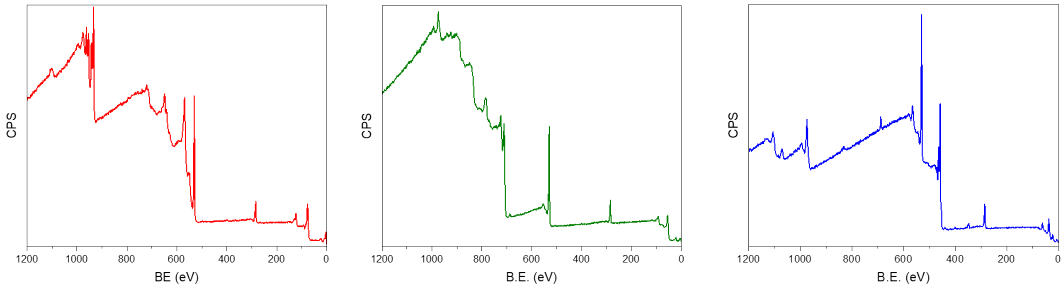

Figure S3: Survey XPS measurements following self-assembly of F-NHC on CuOx, FeOx and TiOx (red, green and blue colored spectra, respectively).

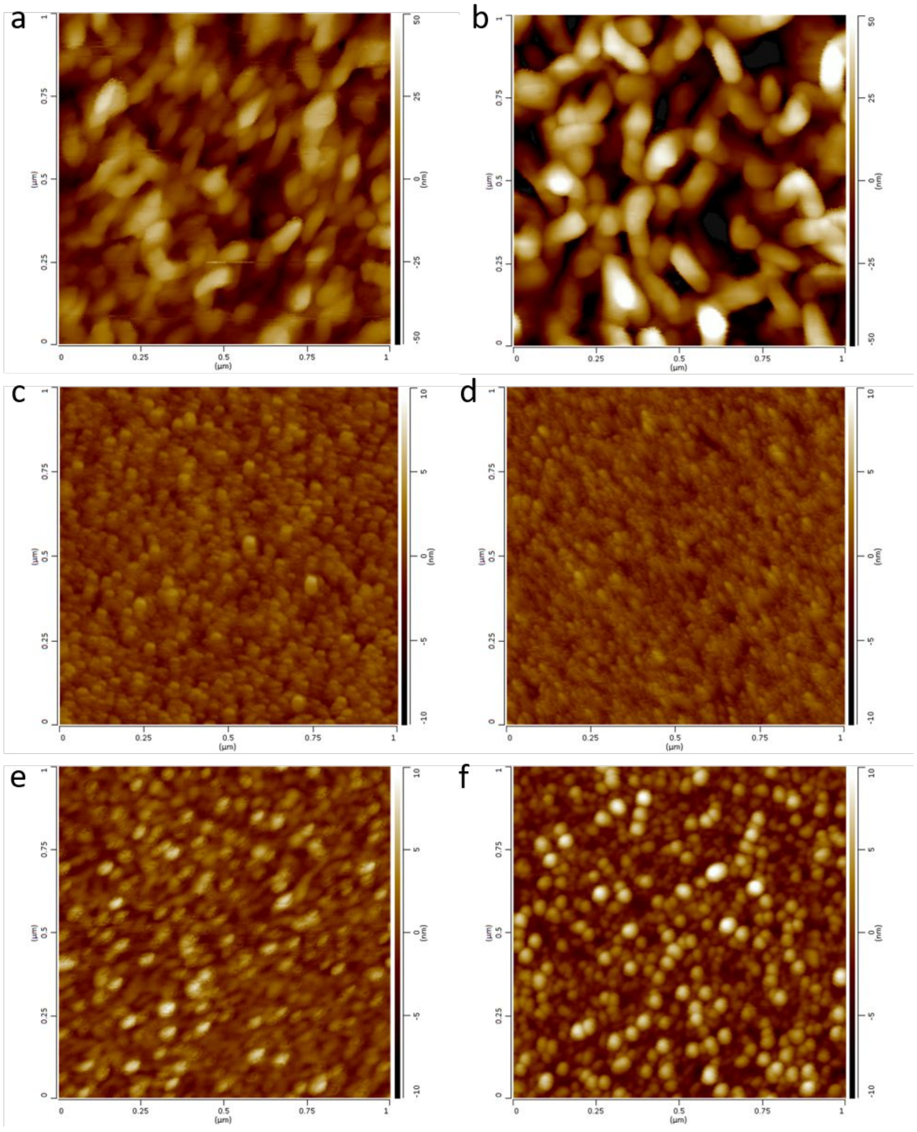

Figure S4: AFM topography of CuOx (a-b), FeOx (c-d) and TiOx (e-f). AFM images were acquired before (a, c and e) and after (b, d, and f) F-NHC deposition.

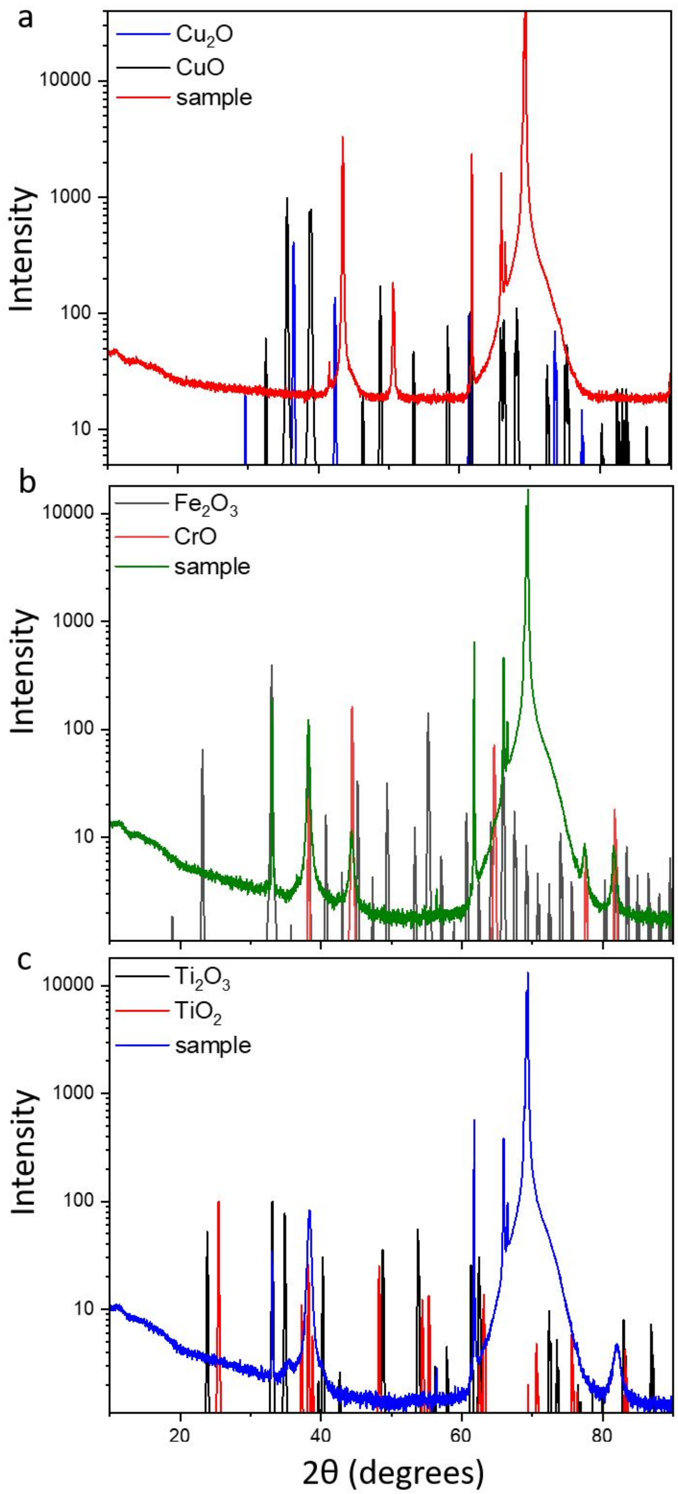

Figure S5: XRD spectra of CuOx (a), FeOx (b) and TiOx (c).

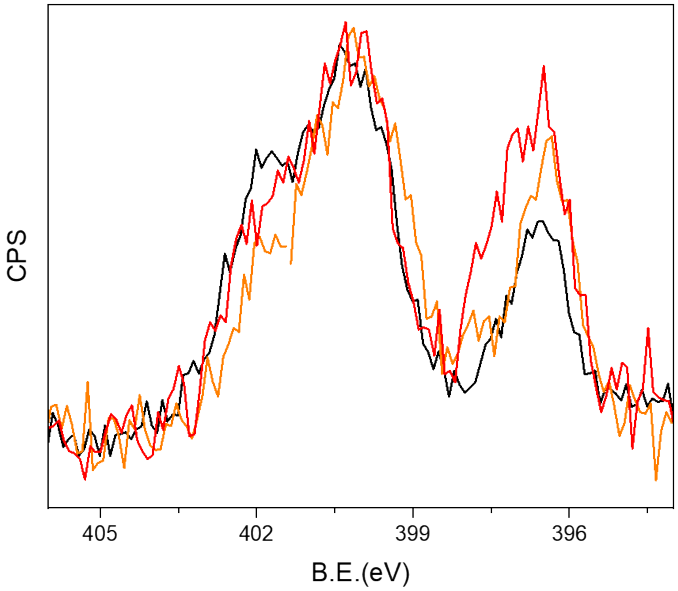

Figure S6: TiOx sample at room temperature (black colored spectrum), and after annealing to 100 (orange colored spectrum) and 200 °C (red colored spectrum) under UHV conditions.

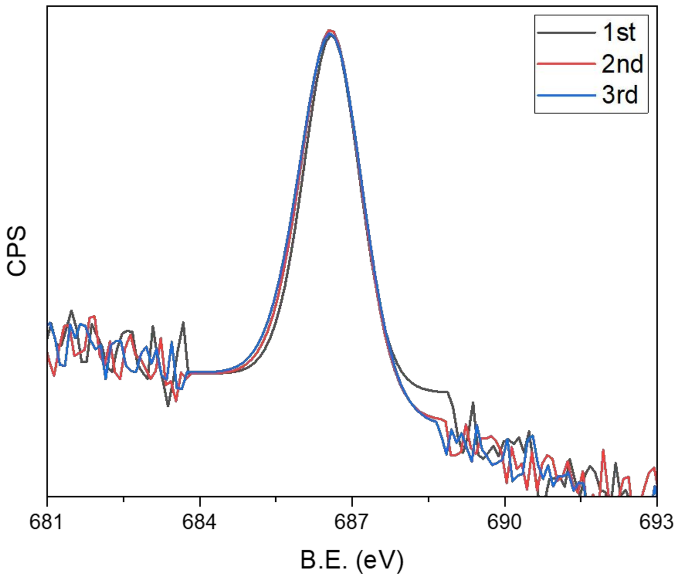

Figure S7: Consecutive F1s XPS spectra of F-NHC on CuOx

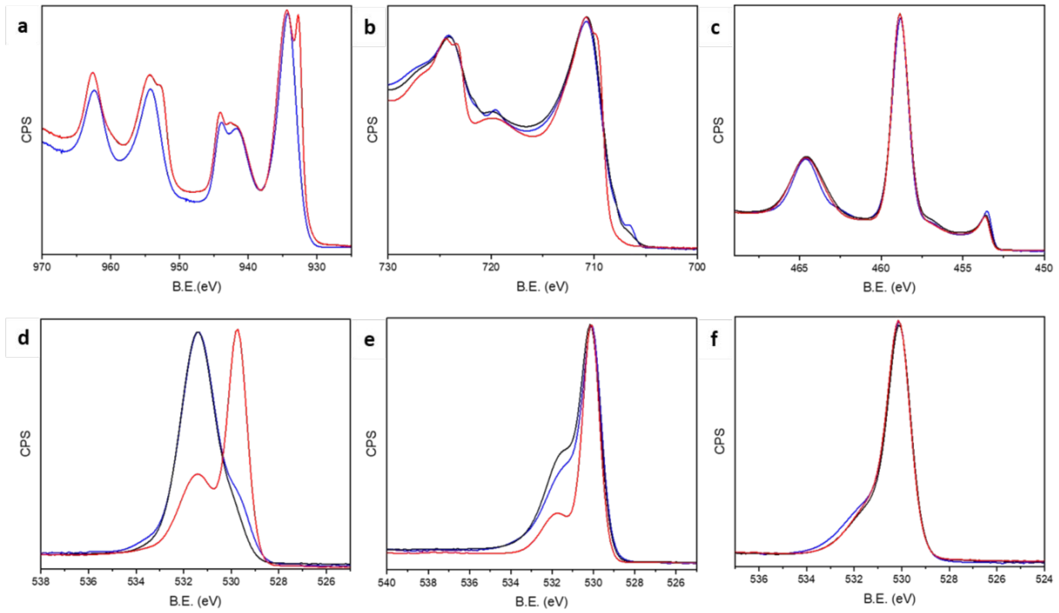

Figure S8: XPS signals of Cu2p (a), Fe2p (b), and Ti2p (c) and O1s XPS signals of CuOx (d) FeOx (e), and TiOx (f) following F-NHC deposition (black), after annealing to 100 °C (blue), and 200 °C (red).

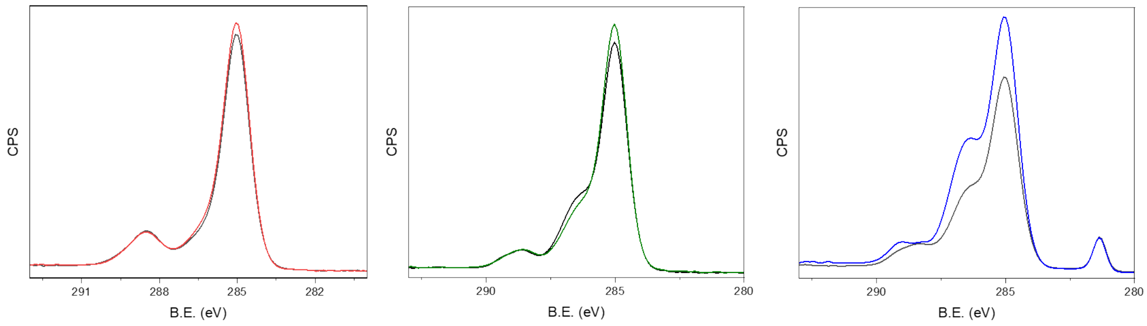

Figure S9: C1s XPS spectra before (black-colored) and after F-NHC deposition on CuOx, FeOx and TiOx (red-, green- and blue-colored, respectively).

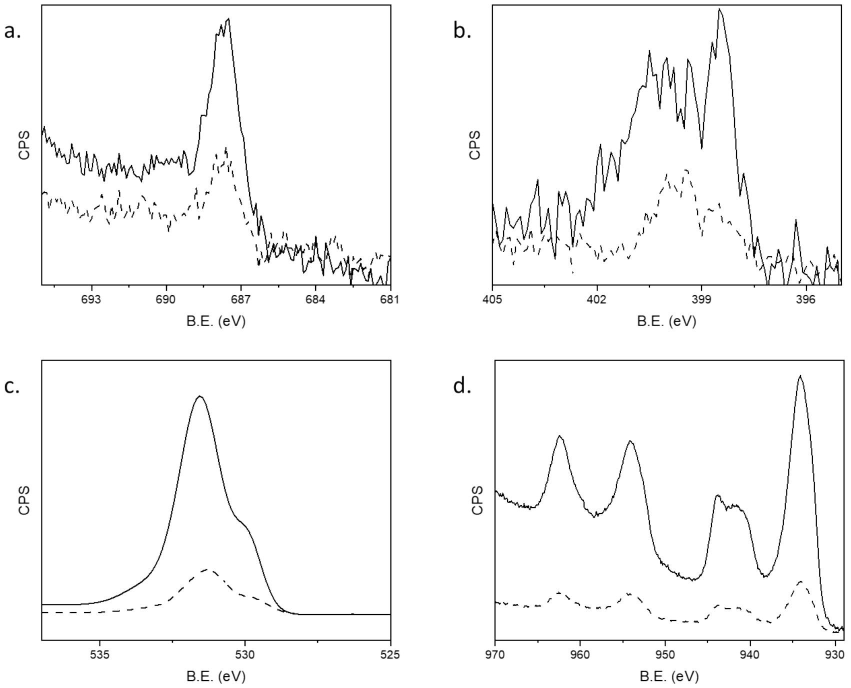

Figure S10: F1s (a), N1s (b), O1s (c), and Cu2p (d) XPS measurements of CuOx film with F-NHC monolayer at a normal (solid line) and a grazing angle (dashed line).

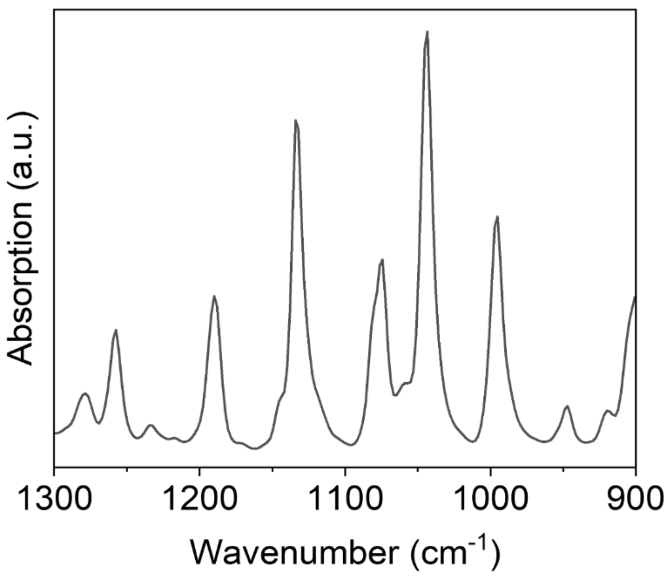

Figure S11: ATR spectrum of the imidazolium precursor of F-NHC

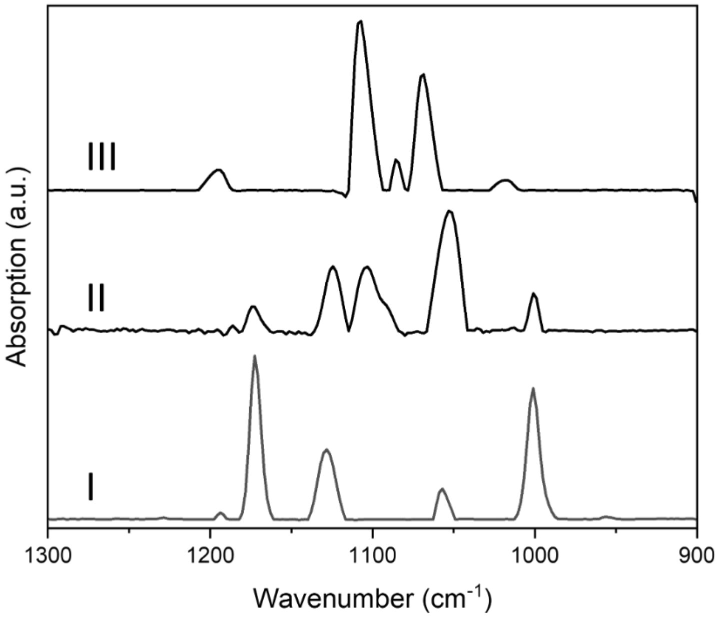

Figure S12: PM-IRRAS spectra of F-NHC monolayers on CuO at room temperature (I), after annealing to 100 (II), and 200 °C (III)

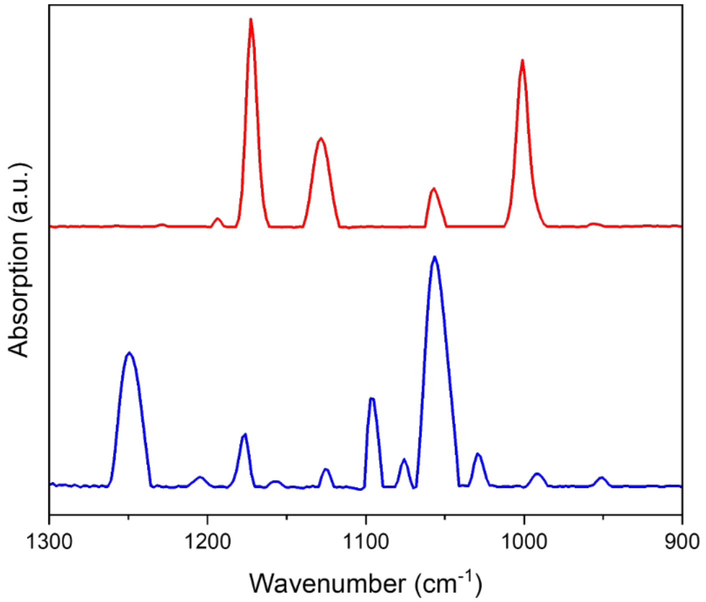

Figure S13: PM-IRRAS signal of dimethyl benzimidazolium iodide based monolayer and F-NHC monolayer (blue and red colored spectra, respectively) that were electrodeposited on CuO_x film.

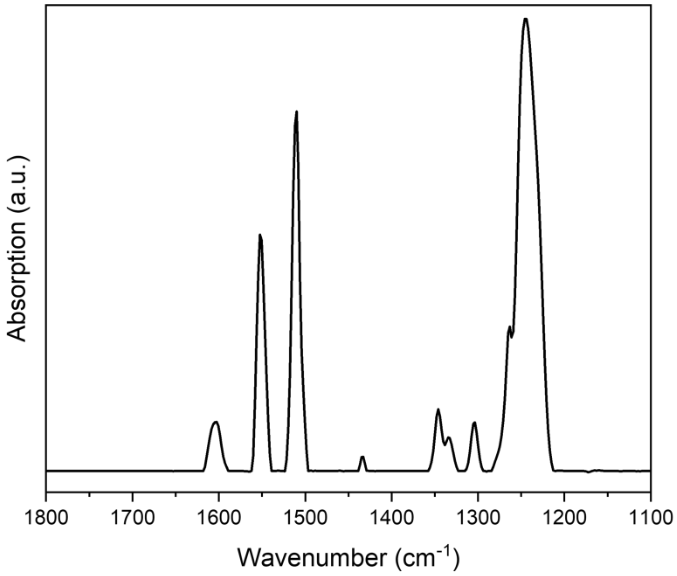

Figure S14: PM-IRRAS spectrum of F-NHC monolayer on Au film.

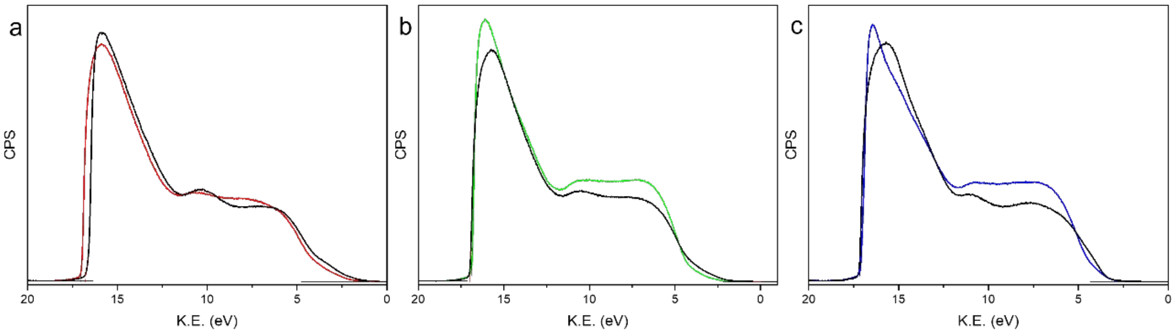

Figure S15: UPS signals of CuO_x (a), FeO_x (b), and TiO_x (c) before (black-coloured) and after (red, green and blue coloured, respectively) self-assembly of F-NHC.
